# Derivation of Mesenchymal Stem Cells through Sequential Presentation of Growth Factors via Gelatin Microparticles in Pluripotent Stem Cell Spheroids

**DOI:** 10.34133/bmr.0184

**Published:** 2025-04-29

**Authors:** Nityanand Prakash, Young Cha, Won-Gun Koh, Hansoo Park, Alvin Bacero Bello, Soo-Hong Lee

**Affiliations:** ^1^ Department of Biomedical Engineering, Dongguk University, Seoul 04620, Republic of Korea.; ^2^ Molecular Neurobiology Laboratory, McLean Hospital and Department of Psychiatry, Harvard Medical School, Belmont, MA 02478, USA.; ^3^ Department of Chemical and Biomolecular Engineering, Yonsei University, Seoul 03722, Republic of Korea.; ^4^ School of Integrative Engineering, Chung-Ang University, Seoul 06911, Republic of Korea.

## Abstract

The use of mesenchymal stem cells (MSCs) in regenerative medicine has gained considerable attention in recent years with the development of clinically relevant MSCs from induced pluripotent stem cells (iPSCs) and embryonic stem cells. Through sequential presentations of appropriate growth factors (GFs), iPSCs can be differentiated into mesodermal cells and then into MSCs. Furthermore, the formation of 3-dimensional cell spheroids, known as embryoid bodies, can be used to mimic in vivo conditions. However, the compact nature of embryoid bodies restricts the efficient and uniform delivery of GFs, leading to the formation of necrotic zones and hindered differentiation. To address this, we developed 2 types of gelatin microparticles (GelMPs) with distinct degradation rates for sequential delivery of GFs to enhance differentiation while preventing necrotic zones. In 2-dimensional culture, bone morphogenetic protein-4 (BMP4) and fibroblast growth factor 2 (FGF2) were identified as key proteins inducing iPSC differentiation into mesodermal cells and MSCs. The sequential presentation of these GFs was optimized for a 3-dimensional culture system by engineering fast-degrading GelMPs conjugated with BMP4 and slow-degrading GelMPs conjugated with FGF2. Our approach facilitated efficient iPSC differentiation into induced mesenchymal stem cells (iMSCs), as demonstrated by enhanced expression of mesodermal markers during the early stages of differentiation and MSC-specific markers at later stages. The resulting iMSCs exhibited characteristic surface markers (e.g., CD73, CD90, CD105, and CD44) and trilineage differentiation capability and were genetically stable. Compared to adult-derived MSCs, iMSCs showed superior proliferative capacity and reduced senescence, making them advantageous for cell therapy and regenerative medicine. This innovative approach of generating iMSCs has vast potential for therapeutic applications.

## Introduction

Mesenchymal stem cells (MSCs) are well-known for their multipotency and paracrine roles in the human body, making them a valuable source of cells used in regenerative medicine [1,2]. To date, various sources of MSCs have been identified and utilized in over 1,300 clinical trials [3–5]. Despite their numerous advantages, adult-derived MSCs are limited by restricted proliferation, inadequate supply, donor variation, and inconsistencies in cell sources [2,6]. To overcome these challenges, the field is devoting significant focus on utilizing pluripotent stem cells (PSCs) to generate clinically relevant MSCs [7]. PSCs possess unlimited proliferative capacity and can differentiate into multiple cell types, potentially making them a reliable source of MSCs.

One approach to differentiate PSCs into MSCs involves the use of mouse OP9 cells, followed by sorting CD73+ cells and maintaining them in MSC media [8]. Despite its selectivity, this method raises concerns about mouse cell contamination and ethical issues associated with embryonic stem cells. Various growth factors (GFs), including bone morphogenetic protein-4 (BMP4), activin A, fibroblast growth factor 2 (FGF2), and epidermal GF, and small molecules like transforming growth factor beta 1 inhibitors (SB431542 or A83-01) and CHIR99021 have also been employed for induced MSC (iMSC) generation [9–11]. Although these factors are effective in promoting differentiation, 2-dimensional (2D) methods are limited in scalability and require substantial amounts of GFs. Additionally, 2D approaches necessitate precise timing and control in presenting various GFs for efficient differentiation, with pre-induction into mesodermal cells followed by differentiation into MSCs proving particularly effective [12]. Genetic engineering of various genes, such as MSX2 and HOX2, has also been explored, but this approach can lead to permanent and undesirable genetic alterations [13,14]. Additionally, biomaterials like fibronectin, gelatin, fibrin, and collagen have been used to culture human PSCs in MSC maintenance media to obtain iMSCs [15–17]. While these approaches provide sufficient extracellular matrix support, they are time-consuming and costly for MSC generation.

To develop an optimal method for generating MSCs from iPSCs, a comprehensive understanding of MSC developmental biology is essential. MSC development begins with the formation of the primitive streak, followed by mesoderm lineage formation and the generation of MSCs through epithelial-to-mesenchymal transition [18]. During this process, key GFs such as BMP4, activin, FGF2, and epidermal GF are produced and utilized by cells in timed and controlled manners to facilitate effective differentiation into MSCs. Additionally, a 3-dimensional (3D) environment is critical for cell growth and development, as it more accurately mimics in vivo conditions [19,20]. Compared to 2D cultures, 3D spheroid cultures enhance cell-to-cell and cell-to-matrix interactions, thereby increasing the release of GFs, cytokines, and extracellular matrix components to ultimately facilitate cell proliferation [21]. However, differentiating cells in 3D conditions is challenging due to the limited and uneven distribution of GFs within spheroids caused by variable diffusion. This can result in a heterogeneous cell population and necessitate continuous supplementation of GFs, thereby limiting scalability.

To address these challenges, various biomaterials have been employed to facilitate the effective delivery of GFs to cell spheroids and enhance their differentiation. For example, microparticles (MPs) made with poly(lactic-*co*-glycolic acid), gelatin, and collagen have shown significant promise for this application [22–24]. Among them, gelatin MPs (GelMPs) are particularly favorable due to their biocompatibility, biodegradability, ease of fabrication, cost-effectiveness, natural origin, and ability to conjugate GFs for sequential delivery required for optimal cell differentiation [25,26].

In this study, we report an innovative one-step 3D cell spheroid system designed to generate iMSCs by mimicking MSC developmental biology via mesoderm lineage induction [18]. We developed 2 types of GelMPs with distinct degradation profiles. Specifically, BMP4 and FGF2 were conjugated to fast-degrading and slow-degrading GelMPs, respectively, to enable their sequential release needed for stepwise mesoderm induction and subsequent MSC differentiation (Fig. S1). This sequential GF-releasing approach notably enhanced iMSC differentiation, as demonstrated by gene and protein expression analyses. Moreover, the incorporation of GelMPs into the cell spheroids reduced cell death, further demonstrating their utility for this application. The obtained iMSCs exhibited characteristics similar to those of adult-derived MSCs and demonstrated trilineage differentiation capability. Importantly, these iMSCs displayed a superior proliferative capacity and reduced senescence compared to adult MSCs, underscoring their potential for applications in cell therapy and regenerative medicine.

## Materials and Methods

### Cell culture and spheroid formation

Human iPSCs were first cultured on a Matrigel-coated plate with the E8 medium (Life Technologies, Korea). At 70% to 80% confluency, the cells were dissociated and obtained as single cells using a gentle cell dissociation reagent (STEMCELL, Korea). These cells were then cultured as monolayers or spheroids.

For the monolayer culture, iPSCs were seeded on a Matrigel-coated plate with the essential E8 medium. After 24 h, the medium was replaced with the MSC differentiation medium—Dulbecco’s modified Eagle medium/F12 (Gibco, Thermo Fisher Scientific, USA) supplemented with 10% knockout serum replacement (Gibco, Thermo Fisher Scientific, USA) (Gibco, Thermo Fisher Scientific, USA), 1% 2-mercaptoethanol (Gibco, Thermo Fisher Scientific, USA), and 1% penicillin–streptomycin antibiotics (Gibco, Thermo Fisher Scientific, USA). The cells were treated with appropriate concentrations of GFs (BMP4 and FGF2).

For the 3D culture, the dissociated cells were seeded in a StemFit microwell (Microfit, Korea) with 3,000 cells/well. The microwell was then centrifuged at 400 × g for 5 min. Embryoid bodies (EBs) formed after 1 d of culture were transferred to ultralow-attachment plates and maintained in the aforementioned differentiation medium.

Following differentiation, immature iMSCs were maintained on 0.1% gelatin-coated plates in low-glucose Dulbecco’s modified Eagle medium (HyClone, Cytiva, USA) supplemented with 10% fetal bovine serum (FBS) (HyClone, Cytiva, USA) and 1% antibiotics, along with adult-derived MSCs (bone-marrow-derived mesenchymal stem cells [BMMSCs] and adipocyte-derived mesenchymal stem cells [ASCs]).

### GelMP fabrication and characterization

GelMPs were formulated by a conventional water-in-oil emulsion method as previously described [24]. Briefly, 5 g of Gelatin Type A (175 Bloom, Electron Microscopy Sciences, Pennsylvania) was fully dissolved in distilled water at 40 to 45 °C until no insoluble particles were observed. Next, 250 ml of olive oil (Sigma-Aldrich, Germany) containing 1% Span 80 (Sigma-Aldrich, Germany) was stirred at 300 rpm, and the gelatin solution was added dropwise using an 18-gauge plastic syringe. The solution was stirred for 1 h at room temperature (RT), followed by an additional 30 min of stirring over ice. Next, 4 or 12 mM glutaraldehyde solution (Sigma-Aldrich, Germany) was added to the solution while stirring. The mixture was then stirred for an additional 2 h to form fast- or slow-degrading GelMPs. Lastly, the obtained MPs were washed with cold acetone to remove oil. The MPs were kept in a humid dryer until usage.

Dry GelMPs were weighed and resuspended in distilled water in preweighed Eppendorf tubes and then incubated overnight with constant agitation. Upon swelling, the tubes were spun at 8,000 rpm for 5 min, and excess water was removed. The swollen particles were weighed. The swelling ratio (*q*) was calculated using Eq. 1:

$$q=\frac{\text{swollen}\;\mathrm{MP}\;\text{weight}\;\left(\mathrm{Ws}\right)}{\mathrm{dry}\;\mathrm{MP}\;\text{weight}\;\left(\mathrm{Wd}\right)}$$ (1)

Finally, the water content was calculated using Eq. 2:

$$\frac{\mathrm{Ws}-\mathrm{Wd}}{\mathrm{Ws}}*100$$ (2)

The size of the obtained GelMPs was determined by scanning electron microscopy analysis. For the degradation assay, 5 mg of wet MPs was first incubated in 500 μl of collagenase type I in phosphate-buffered saline (PBS; 5 μg/ml). Next, 400 μl of supernatant was collected at predetermined time points, and the displaced volume was replenished. The protein concentration was determined using the bicinchoninic acid assay and plotted against time. GelMPs incubated in PBS served as the negative control.

### GF-conjugated GelMP immunostaining

Immunostaining was performed to confirm GF conjugation to GelMPs. The slow- and fast-degrading GelMPs were incubated with primary antibodies against FGF2 (Santa Cruz Biotechnology, USA) and BMP4 (PeproTech, USA) overnight and labeled with Alexa Fluor 488 (Invitrogen, Thermo Fisher Scientific, USA) and Texas Red (Invitrogen, Thermo Fisher Scientific, USA) at 1 μl/100 μl of PBS, respectively. The stained GelMPs were analyzed by fluorescence microscopy.

### GF conjugation

BMP4 (PeproTech, USA) and FGF2 (PeproTech, USA) were conjugated to fast- and slow-degrading GelMPs, respectively, via carbodiimide 1-ethyl-3-(3-dimethylaminopropyl)carbodiimide (EDC)/*N*-hydroxysuccinimide (NHS) chemistry as previously described [24]. Briefly, prewet GelMPs were suspended in 0.05 M 2-(*N*-morpholino)ethanesulfonic acid (MES) buffer (Merck, Sigma-Aldrich, Germany) for 1 h and washed. Next, 4.8 mM EDC (Sigma-Aldrich, Germany) and 48 mM NHS (Sigma-Aldrich, Germany) dissolved in MES buffer were added to the GelMPs solution. After incubation for 1 h at 4 °C, GelMPs were washed again. BMP4 at a concentration of 200 ng/10,000 MPs and FGF2 at a concentration of 140 ng/10,000 MPs was incubated with fast- and slow-degrading GelMPs, respectively, for 24 h at 4 °C with agitation. The MPs were washed with PBS, lyophilized, and sterilized with ethylene oxide before use.

### RNA isolation and qPCR analysis

Total RNA was isolated using the TRIzol reagent (Ambion, Life Technologies, USA) following the manufacturer’s protocol. Messenger RNA was reverse-transcribed into complementary DNA (cDNA) using the TOPscript cDNA Synthesis Kit (Enzynomics, South Korea). Quantitative real-time polymerase chain reaction (qPCR) analysis was performed using the Power SYBR Green PCR Master Mix (Applied Biosystems, UK) with the StepOnePlus Real-Time PCR System (Applied Biosystems, UK) following the manufacturer’s instructions. Target gene expression was normalized to the housekeeping gene 18S for quantification. The primer sequences used for analysis are shown in the Table.

**Table.** qRT-PCR analysis genes’ primers

Gene	Forward primer (5′–3′)	Reverse primer (5′–3′)
Brachyury	GCAAAAGCTTTCCTTGATGC	ATGAGGATTTGCAGGTGGAC
CDX2	GGCTGGAGCTGGAGAAGGAG	TGCGGTTCTGAAACCAGATTTT
TWIST1	GGTATAAGAGCCTCCAAGTCTGC	AAAAGAAAGCGCCCAACGG
GATA1	ATCACACTGAGCTTGCCACA	CAGGCCAGGGAACTCCA
SNAI1	TTCCAGCAGCCCTACGACCAG	GCCTTTCCCACTGTCCTCATC
TBX6	GTCAGAAGCTGTCGGACTCAC	TCCAGTTTAGGGGTGTCCAG
N-CAD	AGCTTCTCACGGCATACACC	GTGCATGAAGGACAGCCTCT
FOXA2	TCCTTGGAATCAAATGGGTCT	TCTGTTAGGATAGTGCGTGG
GATA3	CCACCCCATCACCACCTACC	ACTCCCTGCCTTCTGTGCTG
PAX6	GTCCATCTTTGCTTGGGAAA	TAGCCAGGTTGCGAAGAACT
NANOG	CATGAGTGTGGATCCAGCTTG	TGAGGCATCTCAGCAGAAGAC
SOX2	GGATAAGTACACGCTGCCCG	ATGTGCGCGTAACTGTCCAT
CD73	CAGTACCAGGGCACTATCTGG	AGTGGCCCCTTTGCTTTAAT
CD90	AGAGACTTGGATGAGGAG	CTGAGAATGCTGGAGATG
CD105	CCACTAGCCAGGTCTCGAAG	GATGCAGGAAGACACTGCTG
CD44	CCAGAAGGAACAGTGGTTTGGC	ACTGTCCTCTGGGCTTGGTGTT
PPARγ	GATACACTGTCTGCAAACATATCACAA	CCACGGAGCTGATCCCAA
C/EBPβ	GCA AGA GCC GCG ACA AG	GGC TCG GGC AGC TGC TT
FABP4	GCA TGG CCA AAC CTA ACA TGA	CCT GGC CCA GTA TGA AGG AAA
RUNX2	CAG ACC AGC AGC ACT CCA TA	CAG CGT CAA CAC CAT CAT TC
ALP	GAC AAG AAG CCC TTC ACT GC	AGA CTG CGC CTG GTA GTT GT
OSX	GGG ACT GGA GCC ATA GTG AA	CTC AGC TCT CTC CAT CTG CC
COL2A1	GGGAGTAATGCAAGGACCA	ATCATCACCAGGCTTTCCAG
SOX9	GTA CCC GCA CTT GCA CAA C	TCT CGC TCT CGT TCA GAA GTC
AGG	GCC TGC GCT CCA ATG ACT	ATG GAA CAC GAT GCC TTT CAC

qRT-PCR, quantitative real-time polymerase chain reaction; CDX2, caudal-type homeobox 2; TWIST1, Twist family bHLH transcription factor 1; SNAI1, Snail family transcriptional repressor 1; TBX6, T-box transcription factor 6; N-CAD, neural cadherin; FOXA2, Forkhead box A2; PAX6, paired box 6; SOX2, SRY-box transcription factor 2; PPARγ, peroxisome proliferator-activated receptor gamma; C/EBPβ, CCAAT/enhancer binding protein beta; FABP4, fatty acid binding protein 4; RUNX2, Runt-related transcription factor 2; ALP, alkaline phosphatase; OSX, Osterix; COL2A1; collagen, type II, alpha 1 chain; SOX9, SRY-box transcription factor 9; AGG, aggrecan.

### Western blot analysis

Total protein was extracted from cells using radioimmunoprecipitation assay lysis buffer (Sigma, USA). Protein concentrations were quantified via the bicinchoninic acid assay, and 20 ng/μl protein per sample was separated via denaturing polyacrylamide gel electrophoresis and transferred to a nitrocellulose membrane. The membrane was blocked with 5% nonfat skimmed milk (BD Difco, USA) in TBST solution (Tris-buffered saline with Tween 20) for 1 h and subsequently incubated with primary antibodies (1:500 dilution) against Brachyury, Snail family transcriptional repressor 1 (SNAI1), SRY-box transcription factor 2 (SOX2), NANOG, CD73, CD44, CD105, peroxisome proliferator-activated receptor gamma (PPARγ), and SRY-box transcription factor 9 (SOX9), and Runt-related transcription factor 2 (RUNX2) in TBST solution with 5% bovine serum albumin overnight at 4 °C. The membrane was then washed, reblocked, and incubated with secondary antibodies (1:2,500 dilution) against goat anti-mouse-horseradish peroxidase or goat anti-rabbit-horseradish peroxidase in 5% nonfat skimmed milk for 1 h. Immunoreactive bands were visualized using the ECL Select reagent (GE Healthcare, USA) and Bio-Rad Image Lab software. All blots derived from the same experiment were processed simultaneously.

### FACS analysis

Cell surface antigens were assessed by fluorescence-activated cell sorting (FACS) analysis. Cells were dissociated with 0.05% trypsin/EDTA (HyClone, Cytiva, USA), washed with PBS, blocked with FACS buffer (2% FBS), and incubated for 30 min. The samples were washed then with 2% FBS and stained with antibodies against human CD73 (CD73–phycoerythrin [PE], BioLegend, 1:100), CD90 (CD90–PE, BioLegend, 1:100), CD44 (CD44–PE, BioLegend, 1:100), CD34 (CD34–PE, BioLegend, 1:100), CD45 (CD45–PE, BioLegend, 1:100), and stage-specific embryonic antigen 4 (SSEA4) (SSEA4–PE, BioLegend, 1:100) for 30 min at 4 °C in the dark. The corresponding mouse/rabbit isotype antibodies (Abcam, 1:100) were used as controls. The samples were washed 3 times with FACS buffer and analyzed using an Accuri C6 flow cytometer (BD Biosciences). The samples were stained with propidium iodide staining for 15 min to sort dead cells.

### H&E staining

To visualize GelMPs embedded within cell spheroids, hematoxylin and eosin (H&E) staining was performed. Spheroids were collected, washed with PBS, and fixed with 4% paraformaldehyde. After 1 h, spheroids were washed with PBS, embedded in HistoGel, and sectioned. The sections had been rehydrated before H&E staining was performed. The stained sections were observed using a light microscope.

### Senescence assay

The senescence of cells was assessed using a β-galactosidase assay kit (Cell Signaling Technology, USA). Cells at passages 5 and 10 were harvested, seeded, cultured for 24 h, and washed with PBS. The cells were fixed and stained following the provided protocol. The cells were incubated with the staining solution for 16 h, and blue coloration in the cells was observed.

### Cell proliferation assay

Cell proliferation was assessed by cumulative growth, doubling time, and the Cell Counting Kit-8 assay. Cumulative growth from passages 4 to 10 was assessed at 4-d intervals. The cells were counted at each passage, and cell doubling was calculated using Eq. 3:

$$\left\{{\text{log}}_{10}\left(\mathrm{Nf}\right)-{\text{log}}_{10}\left(\mathrm{Ni}\right)\right\}/{\text{log}}_{10}\left(2\right)$$ (3)

where Nf represents the final cell count and Ni represents the initial seeding cell count. Cumulative growth was determined by summing the cell doubling values. Cell doubling time was determined by dividing the cell doubling value by the culture duration (days).

### Spheroid viability

The viability of spheroids on day 11 was determined by the live/dead assay. A working solution containing calcein AM and ethidium homodimer in the culture medium was added to the spheroids and incubated for 30 min. Next, the spheroids were imaged by fluorescence microscopy, and the size of the spheroids was measured using ImageJ. The live/dead assay for MSCs was performed with a reduced incubation time of 15 min.

### Enzyme-linked immunosorbent assay

BMP4 and FGF2 release from GelMPs was quantified using enzyme-linked immunosorbent assay (R&D Systems, USA), following the manufacturer’s instructions. The samples and standards were incubated in an enzyme-linked immunosorbent assay plate at RT for 2 h. After washing, 200 μl of BMP4 and FGF2 antibody was added to the respective wells and incubated at RT for another 2 h. The wells were then washed 4 times with wash buffer. The substrate solution was added to each well and incubated at RT for 30 min in the dark. The reaction was terminated by adding 50 μl of stop solution to each well. The optical density at 450 nm was immediately determined using a microplate reader. BMP4 and FGF2 concentrations were calculated based on the constructed standard curve.

### Trilineage differentiation

MSCs were subjected to chondrogenesis, adipogenesis, and osteogenesis, as described previously with minor modifications [27]. The differentiated cells were analyzed by qPCR, western blot (WB), and immunocytochemistry. The differentiation efficiency was analyzed by quantifying the related markers.

### GTG karyotyping analysis

To check for genetic stability postdifferentiation, karyotyping analysis was conducted. The parent iPSCs (hFsiPSC cell line, passage no. 25 [P25]) and 3D generated iMSCs (passage no. 5 [P5]) were cultured in 25-ml T-flasks and were sent to the Gendix company for analysis. Briefly, karyotyping was conducted by analyzing the Giemsa trypsin G-banding (GTG) that involved proteolytic enzyme trypsin pretreatment, staining with Giemsa, and visualization under a bright-field microscope (*n* = 10). Detailed information is shown in Supplementary Materials: P5 iMSC karyotype results and P25 iPSC karyotype results.

### Statistical analysis

All data represent results from 3 independent experiments, each conducted in triplicate. The data are presented as mean ± standard deviation (SD). A *P* value <0.05 was considered statistically significant: ns = not significant, *P < 0.05, **P < 0.01, ***P < 0.001, and ****P < 0.0001. One-way analysis of variance (ANOVA) followed by Tukey’s post hoc test was used to compare mean values among groups. Individual data points and *P* values for significance are indicated in graphs.

## Results

### BMP4 and FGF2 induce the differentiation of iPSCs into iMSCs in 2D culture conditions

**Fig. 1.**
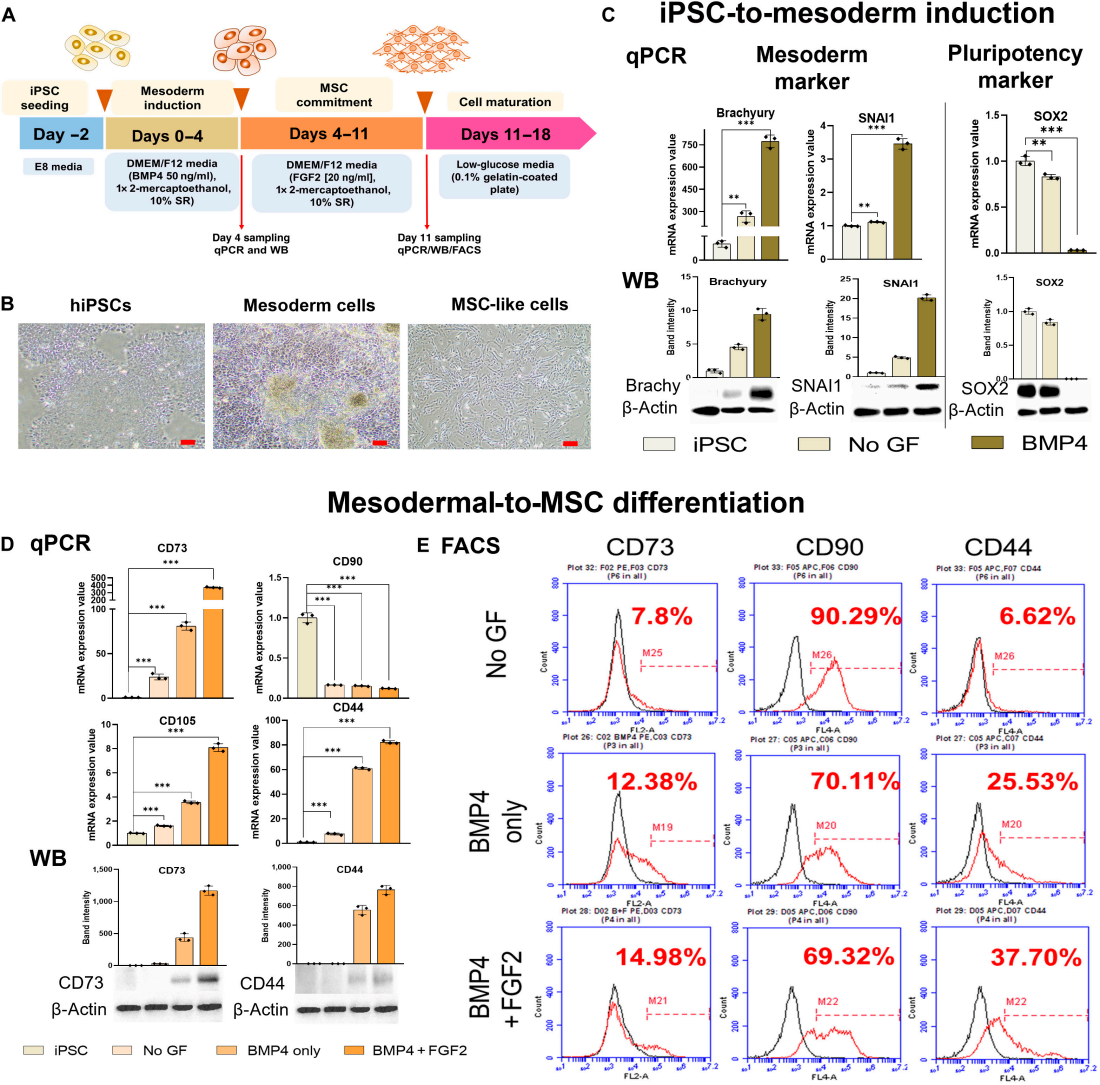
Bone morphogenetic protein-4 (BMP4)- and fibroblast growth factor 2 (FGF2)-induced differentiation of induced pluripotent stem cells (iPSCs) to mesenchymal stem cells (MSCs) in 2-dimensional (2D) conditions. (A) Schematic diagram depicting iPSC-to-induced MSC (iMSC) differentiation via mesoderm induction. (B) Images of iPSCs, mesoderm-induced cells, and iMSCs. Scale bar = 200 μm. (C) Quantitative real-time gene analysis (qPCR; top panel) and western blot (WB) analysis (bottom panel) of the mesoderm markers Brachyury and SNAI1 (Snail1), along with the pluripotency marker SOX2, at 4 d postdifferentiation. (D) qPCR (top panel) and WB analysis (bottom panel) of the MSC markers CD73, CD90, CD105, and CD44 at 11 d postdifferentiation. (E) Flow cytometry (fluorescence-activated cell sorting [FACS]) analysis of the MSC markers CD73, CD90, and CD44 in cells treated with either BMP4 only or BMP4 + FGF2. The qPCR data were normalized to 18S. The WB data were normalized to β-actin. All data represent results from 3 independent experiments, each conducted in triplicate. The data are presented as mean ± SD (ns, not significant; *P < 0.05; **P < 0.01; ***P < 0.001; ****P < 0.0001). Individual data points and significance levels are indicated in graphs. DMEM, Dulbecco’s modified Eagle medium; SR, serum replacement; hiPSCs, human iPSCs; mRNA, messenger RNA; GF, growth factor.

Before establishing the optimal 3D conditions for iPSC differentiation into iMSCs, we first sought to identify the most effective GFs and their optimal concentrations and incubation times for mesoderm induction and MSC differentiation in 2D conditions. Four days of incubation with BMP4 at 50 ng/ml yielded the most efficient mesoderm induction among the tested conditions (Fig. S2). In terms of MSC differentiation, 7 d of incubation with FGF2 at 20 ng/ml, following mesoderm induction, led to notable increases in related markers (Fig. S3). We hypothesized that this procedure efficiently differentiated iPSCs into MSCs by accurately mimicking in vivo conditions [28]. Thus, 4 d of mesoderm induction followed by 7 d of MSC differentiation was established as the optimal timeline (Fig. 1A). Notable morphological changes were observed during both phases (Fig. 1B), with MSCs exhibiting an elongated and adherent morphology characteristic of fibroblasts. Four days of BMP4 supplementation increased the expression of the mesoderm markers Brachyury and SNAI1 while reducing the pluripotency marker SOX2 compared to those of controls (Fig. 1C). Seven days of FGF2 supplementation increased the expression of the MSC markers CD73, CD44, and CD105 (Fig. 1D). These findings were further corroborated by FACS analysis, which revealed increased levels of CD73 and CD44 in the BMP4 + FGF2-treated cells compared to those treated with BMP4 alone (Fig. 1E). This underscored the critical role of FGF2 in the differentiation of iPSCs into MSCs. While FGF2 is commonly recognized for its role in promoting cell proliferation, it has also been effectively utilized to facilitate cell differentiation [12,29–31]. Accordingly, we observed that FGF2 greatly contributes to the differentiation of mesodermal cells into MSCs.

### The 200-μm EBs greatly enhance the induction of mesoderm lineage

**Fig. 2.**
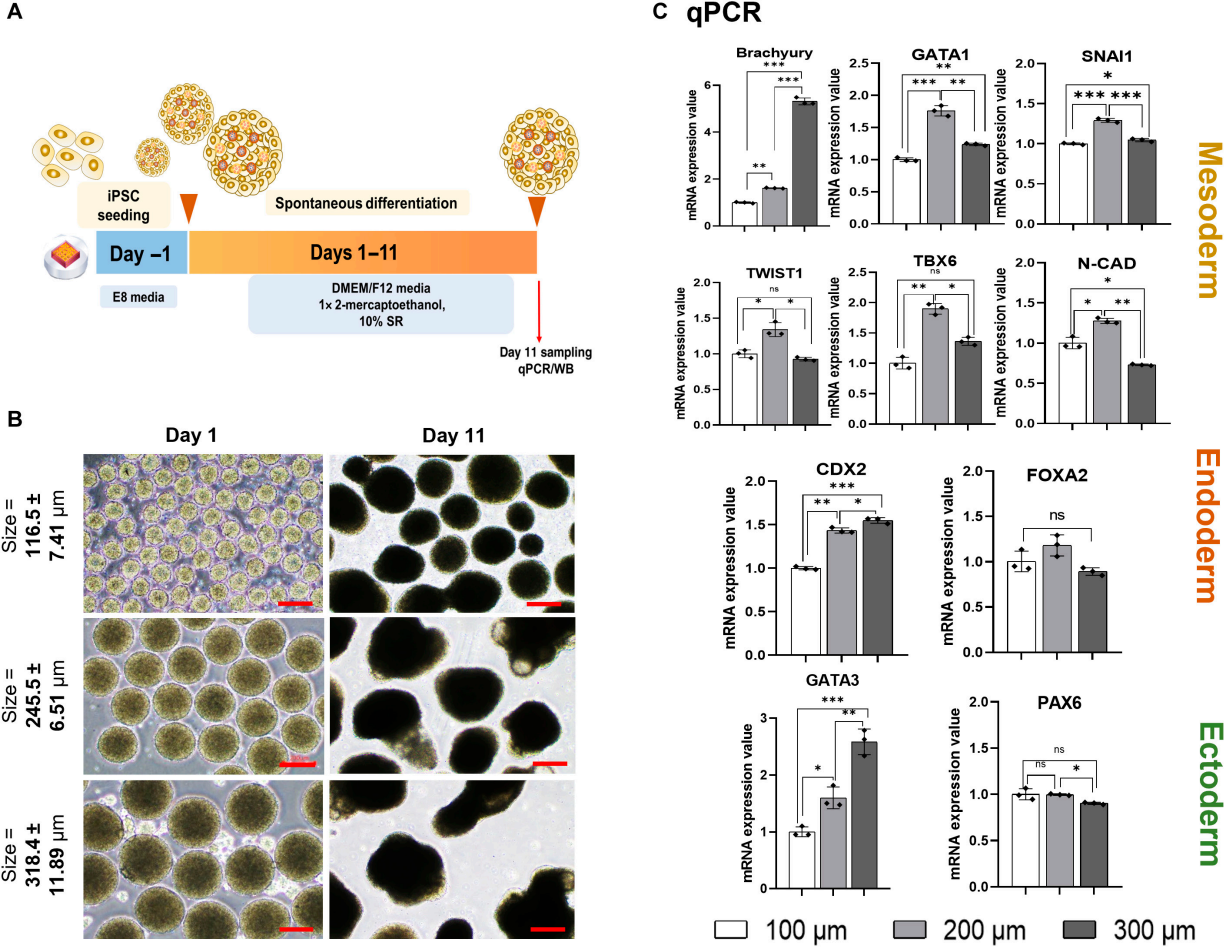
The effects of embryoid body (EB) size on iPSC induction to mesoderm. (A) Schematic diagram depicting EB formation and differentiation. (B) Representative images of EBs in varying sizes—approximately 100, 200, and 300 μm. Images were taken on day 1 and day 11. Scale bar = 200 μm. (C) qPCR analysis of mesoderm markers (Brachyury, SNAI1, Twist1, GATA1, N-Cad, and TBX6), endoderm markers (CDX2 and FOXA2), and ectoderm markers (GATA3 and PAX6) at 11 d postdifferentiation. The qPCR data were normalized to 18S and represent results from 3 independent experiments, each conducted in triplicate. The data are presented as mean ± SD (ns, not significant; *P < 0.05; **P < 0.01; ***P < 0.001; ****P < 0.0001). Individual data points and significance levels are indicated in graphs.

In addition to the availability of GFs, the size of spheroids is critical for directing cell differentiation into the mesodermal lineage in 3D cultures [32–34]. To investigate the impact of this factor, we generated EBs of varying sizes by adjusting the cell seeding density and allowing them to differentiate spontaneously (Fig. 2A). iPSCs were cultured at 1,000, 3,000, and 6,000 cells/well, resulting in EBs of approximately 100, 200, and 300 μm in diameter, respectively (Fig. 2B, left panel).

These EBs were cultured in a spontaneous differentiation medium for 11 d to promote mesoderm, endoderm, and ectoderm differentiation (Fig. 2B, right panel). qPCR analysis revealed that the 200-μm spheroids exhibited considerably increased expression of mesodermal markers (SNAI1, Twist1, neural cadherin [N-cad], and T-box transcription factor 6 [TBX6]) compared to 100- and 300-μm spheroids, except for the early mesodermal marker Brachyury, which showed increased expression in the 300-μm EBs (Fig. 2C, upper panel). This suggested that the mesoderm induction in larger spheroids was delayed. Notably, the ectoderm marker GATA3 and the endoderm marker caudal-type homeobox 2 (CDX2) were also elevated in the 300-μm EBs (Fig. 2C, bottom panel). Collectively, these results indicated that the 200-μm EBs are most effective in promoting mesoderm differentiation under the tested experimental conditions. Thus, these EBs were used for all subsequent experiments.

### Fabrication of fast- and slow-degrading GelMPs for sequential release of GFs

**Fig. 3.**
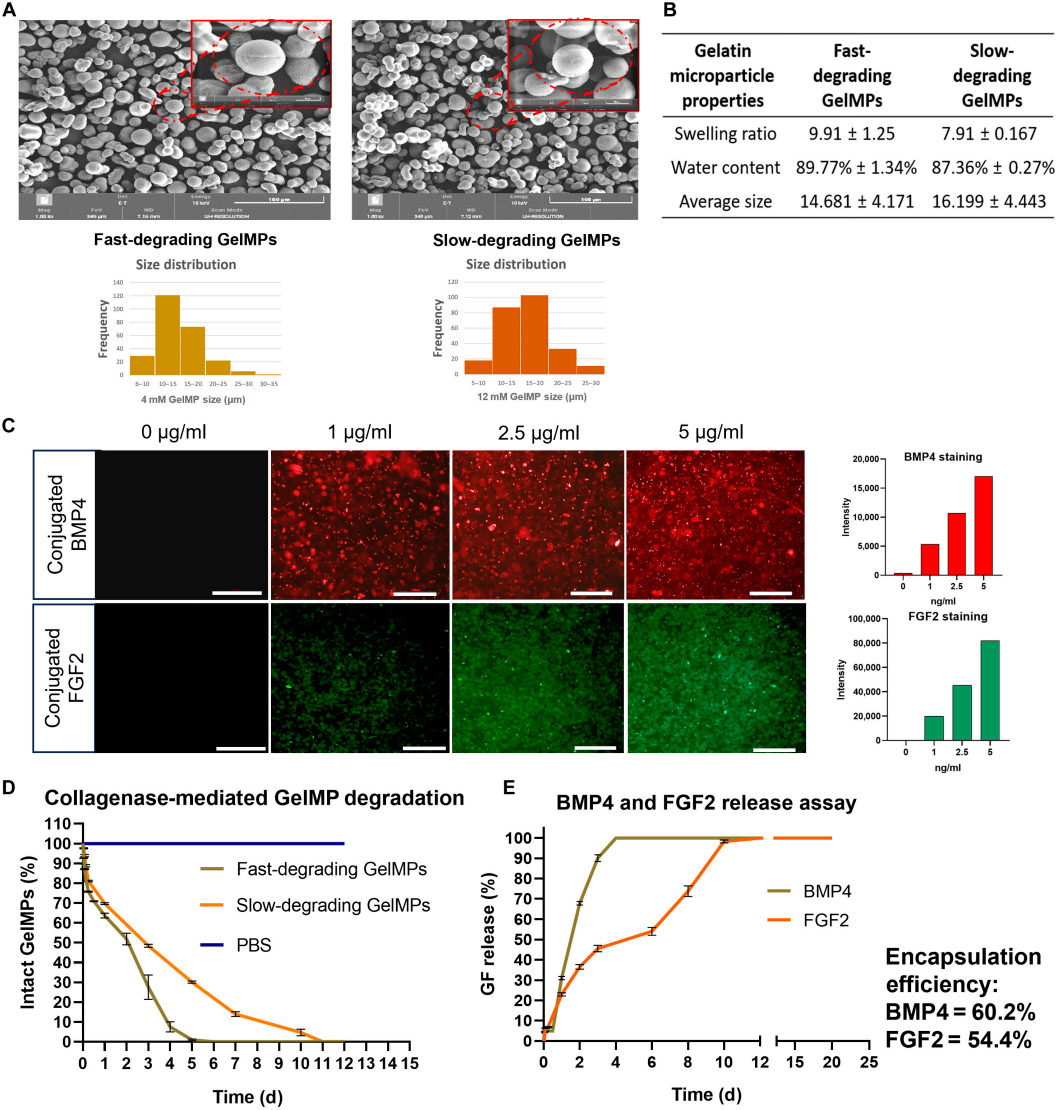
Gelatin microparticle (GelMP) fabrication, characterization, GF conjugation, and the release profiles of BMP4 and FGF2. (A) Scanning electron microscopy (SEM) images of fast-degrading (4 mM glutaraldehyde) and slow-degrading (12 mM glutaraldehyde) GelMPs. Scale bar = 100 μm. Size distributions of fast- and slow-degrading GelMPs. (B) Physical properties of fast- and slow-degrading GelMPs. (C) Representative immunofluorescent images of BMP4-conjugated fast-degrading GelMPs and FGF2-conjugated slow-degrading GelMPs. Scale bar = 100 μm. (D) Degradation rate of 5 mg of microparticles in collagenase (5 μg/ml) solution. (E) Cumulative release (%) of 1 μg of BMP4 from fast-degrading GelMPs and 1 μg of FGF2 from slow-degrading GelMPs incubated for 20 d in collagenase (5 μg/ml) solution. The obtained data represent results from 3 independent experiments, each conducted in triplicate. PBS, phosphate-buffered saline.

GelMPs were formulated using a conventional water-in-oil emulsion method and chemically cross-linked with varying concentrations of glutaraldehyde to control the degradation rate. Fast-degrading GelMPs were achieved at 4 mM glutaraldehyde, and slow-degrading GelMPs, at 12 mM. Scanning electron microscopy analysis (Fig. 3A) indicated that fast-releasing GelMPs have an average diameter of 14.68 ± 4.17 μm, while slow-releasing GelMPs are 16.199 ± 4.44 μm on average.

The physical properties of GelMPs were assessed by incubating them in PBS. The fast-releasing GelMPs showed a water content and a swelling ratio of 89.77% ± 1.34% and 9.91 ± 1.25, respectively, while the slow-releasing GelMPs displayed 87.36% ± 0.27% and 7.91 ± 0.17 (Fig. 3B). Next, varying amounts of BMP4 and FGF2 were conjugated to fast- and slow-degrading GelMPs, respectively, by EDC/NHS chemistry. Immunofluorescence imaging confirmed successful conjugation of these GFs, as evidenced by increasing band intensity with increasing GF concentration (Fig. 3C). This also demonstrated our ability to modulate the ratio of GFs to fit our needs. The degradation rates of the MPs were then evaluated in collagenase type I (5 μg/ml) solution. As expected, the fast-releasing GelMPs degraded within 4 to 5 d of incubation, while the slow-degrading GelMPs took 10 to 11 d to degrade (Fig. 3D). These differences in physical properties between the 2 GelMP types were attributed to variations in their cross-linking degrees, achieved by modulating the glutaraldehyde concentration. The encapsulation efficiencies of BMP4 and FGF2 conjugated to their corresponding GelMPs were 60.2% and 54.4%, respectively. The GF-release assay showed an initial burst release of both GFs upon incubation. BMP4 from fast-degrading GelMPs was released within 4 d of incubation, while FGF2 from slow-degrading GelMPs was released over 11 d (Fig. 3E). These results further demonstrated the utility of our system for sequential and controlled release of GFs. For subsequent differentiation of iPSCs into iMSCs, both MPs are mixed in a ratio of 1:1; only the concentrations of GFs were varied according to the required concentration in the experiment.

### The effects of GelMPs on overall health, viability, and proliferation of spheroids

Spheroids exhibit a 3D spherical morphology with multiple layers of tightly clustered cells. This configuration facilitates cell-to-cell interactions but limits the uniform diffusion of nutrients and oxygen within the spheroids, resulting in cell death in the inner regions near the core [35,36]. MPs are effective in addressing this limitation and preventing the formation of necrotic zones.

**Fig. 4.**
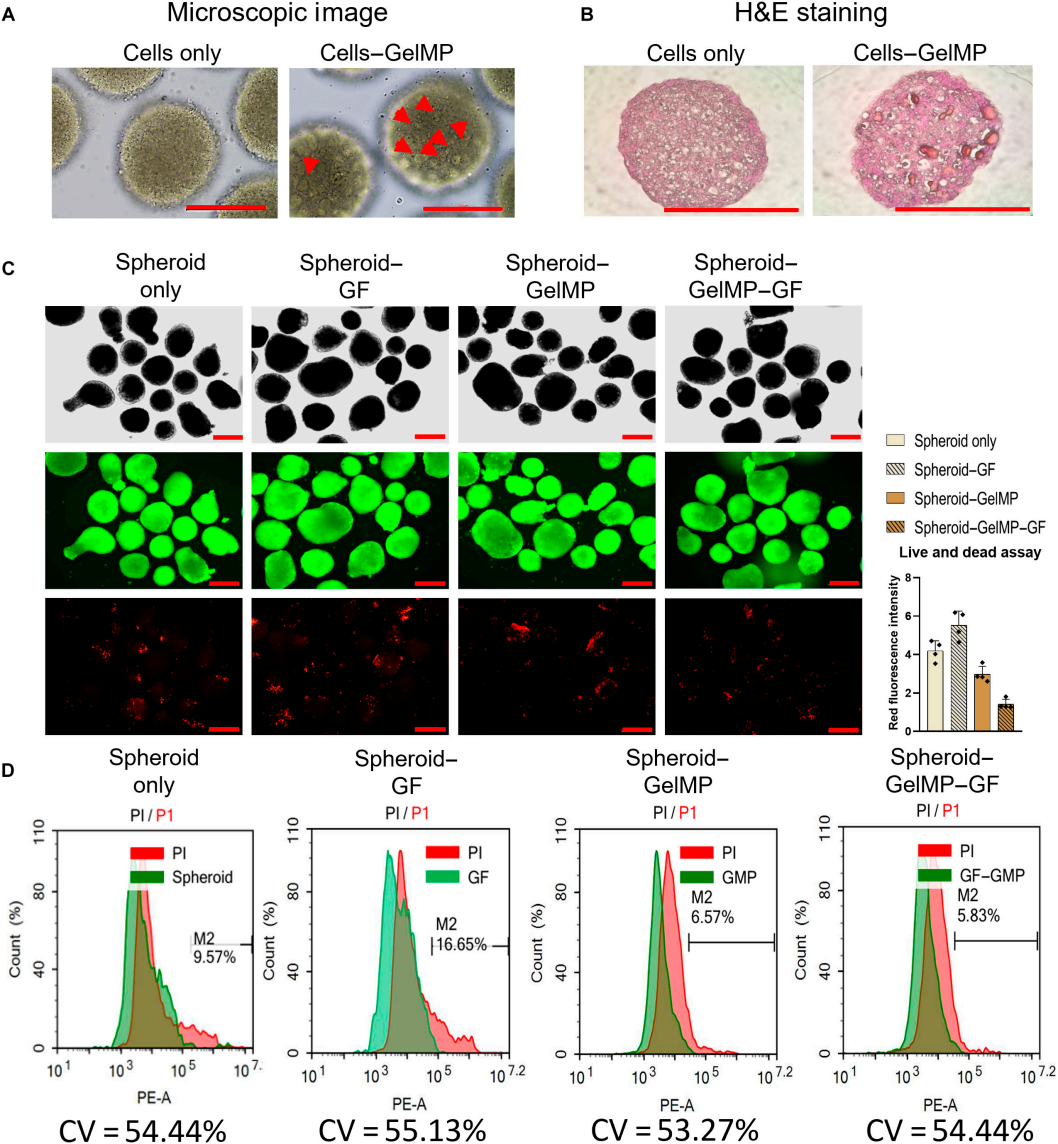
The effects of GelMPs on the overall health and viability of spheroids. (A) Images of GelMP distribution within spheroids on day 1. Red arrows indicate the location of GelMPs. Scale bar = 200 μm. (B) Hematoxylin and eosin (H&E) staining of the sections of spheroids either untreated or treated with GelMPs. Scale bar = 200 μm. (C) Representative live/dead staining of spheroids only, spheroid–GF, spheroid–GelMP, and spheroid–GelMP–GF. Red fluorescence (ethidium bromide) was used to quantify dead cells. Scale bar = 300 μm. (D) FACS analysis of propidium iodide (PI)-stained spheroids only, spheroid–GF, spheroid–GelMP, and spheroid–GelMP–GF. The obtained data represent results from 3 independent experiments, each conducted in triplicate. GelMP, gelatin microparticle; CV, coefficient of variation; PE-A, phycoerythrin–Alexa Fluor 647.

To assess the effects of GelMPs on spheroid formation, they were mixed at a ratio of 1:60 (GelMPs:cells). GelMPs had no impact on the formation or morphology of iPSC spheroids. GelMPs were randomly distributed throughout the spheroids (Fig. 4A, red arrows), as indicated by dark-pink-colored particles in light microscopy images (Fig. 4B). For 3D culture, the following 4 groups were tested: untreated spheroids (spheroid-only), spheroids with soluble GFs (spheroid–GF), spheroids with GelMP (spheroid–GelMP), and spheroids with GF-conjugated GelMPs (spheroid–GelMP–GF). Compared to the spheroid-only and spheroid–GF groups, the spheroid–GelMP and spheroid–GelMP–GF groups showed higher viability, as determined by the live/dead assay (Fig. 4C). Notably, FACS analysis indicated a similar trend, with the presence of GelMPs being associated with a reduction in dead, propidium iodide-stained cells (Fig. 4D). These findings suggest that GelMP–GF occupies space within the EBs to prevent dense cell clustering that can limit nutrient and oxygen circulation in inner regions near the core.

### Generation of 3D iPSC-derived iMSCs via sequential release of GFs from GelMPs

The transition to 3D differentiation approaches has significantly improved the generation of iMSCs compared to those from traditional 2D approaches [37–40]. By mimicking in vivo cellular interactions, 3D differentiation models have significantly enhanced the efficiency and scalability of iMSC production.

**Fig. 5.**
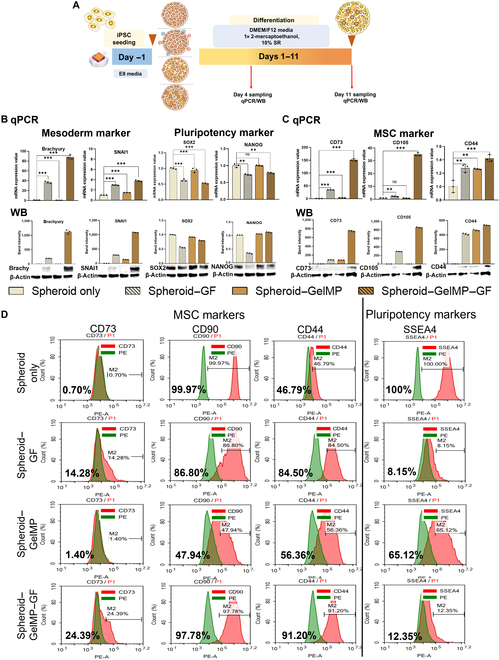
The effects of soluble GFs and GelMP–GF on 3-dimensional (3D) differentiation of iPSCs into MSCs and the generation of relevant markers. (A) Schematic illustration depicting the differentiation of spheroids only, spheroid–GF, spheroid–GelMP, and spheroid–GelMP–GF into MSCs. (B) qPCR (top panel) and WB (bottom panel) analyses of mesoderm markers (Brachyury and SNAI1) and pluripotent markers (SOX2 and NANOG) on day 4. (C) qPCR (top panel) and WB (bottom panel) analyses of MSC markers (CD73, CD105, and CD44) on day 11. (D) FACS analysis of MSC markers (CD73, CD90, and CD44) and the pluripotency marker stage-specific embryonic antigen 4 (SSEA4). The qPCR data were normalized to 18S. The WB data were normalized to β-actin. All data represent results from 3 independent experiments, each conducted in triplicate. The data are presented as mean ± SD (ns, not significant; *P < 0.05; **P < 0.01; ***P < 0.001; ****P < 0.0001). Individual data points and significance levels are indicated in graphs. PE, phycoerythrin.

The optimized 2D culture conditions were adapted for use in 3D spheroid cultures. The impact of GelMP–GF on iMSC generation was assessed against soluble GFs (Fig. 5A). During the initial 4 d of differentiation, the cells were incubated with 200 ng/ml BMP4, followed by incubation with 140 ng/ml FGF2 from days 5 to 11. BMP4 promoted mesoderm induction, as indicated by increased gene expressions of Brachyury and SNAI1 and decreased SOX2 and NANOG (Fig. 5B, top panel). This was further supported by WB analysis (Fig. 5B, bottom panel). Furthermore, FGF2 enhanced MSC differentiation, as indicated by increased expression of CD73, CD105, and CD44 on day 11, observed at both the gene (Fig. 5C, top panel) and protein (Fig. 5C, bottom panel) levels. Notably, both the spheroid–GF and spheroid–GelMP–GF groups showed higher levels of mesoderm and MSC markers compared to the spheroid-only and spheroid–GelMP groups. This indicated that the presence of GFs is essential for MSC differentiation. Interestingly, MP-conjugated GFs were found to be more effective than soluble GFs in promoting MSC differentiation, as indicated by FACS analysis (Fig. 5D), likely due to the enhanced delivery of GFs facilitated by MPs. Apart from this, we observed that there was a decrease in mesoderm markers in the GF-containing group on day 11 and MSC gene expression was not yet observed in any groups on day 4 (Fig. S4). We also mentioned the coefficient of variation in Fig. S5.

### Three-dimensional iMSCs display trilineage differentiation capability comparable to that of adult-derived MSCs

**Fig. 6.**
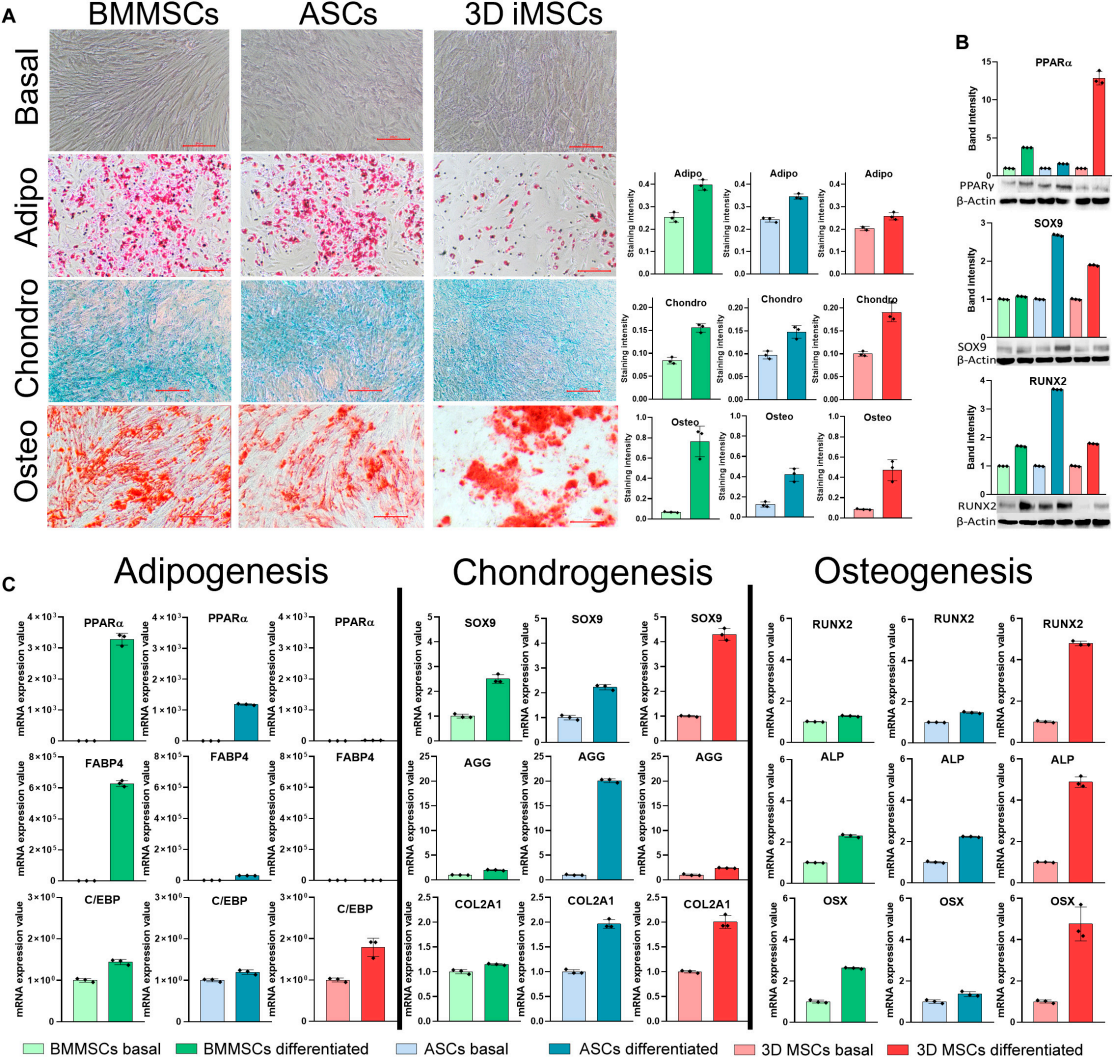
Adipogenic, chondrogenic, and osteogenic differentiation of 3D iMSCs. (A) Oil Red O- (adipogenesis), alcian blue- (chondrogenesis), and alizarin red-stained (osteogenesis) adult MSCs and iMSCs. The relative staining intensities were calculated following stain extraction. Scale bar = 200 μm. (B) WB analysis of the differentiation markers PPARγ (adipogenesis), SOX9 (chondrogenesis), and RUNX2 (osteogenesis). The band intensities were calculated using ImageJ. (C) qPCR analyses of adipogenic (PPARγ, FABP4, and C/EBP), chondrogenic (SOX9, AGG, and COL2A1), and osteogenic markers (RUNX2, ALP, and OSX). The qPCR data were normalized to 18S. The WB data were normalized to β-actin. All data represent results from 3 independent experiments, each conducted in triplicate. The data are presented as mean ± SD (ns, not significant; *P < 0.05; **P < 0.01; ***P < 0.001; ****P < 0.0001). Individual data points and significance levels are indicated in graphs. BMMSCs, bone-marrow-derived mesenchymal stem cells; ASCs, adipocyte-derived mesenchymal stem cells.

**Fig. 7.**
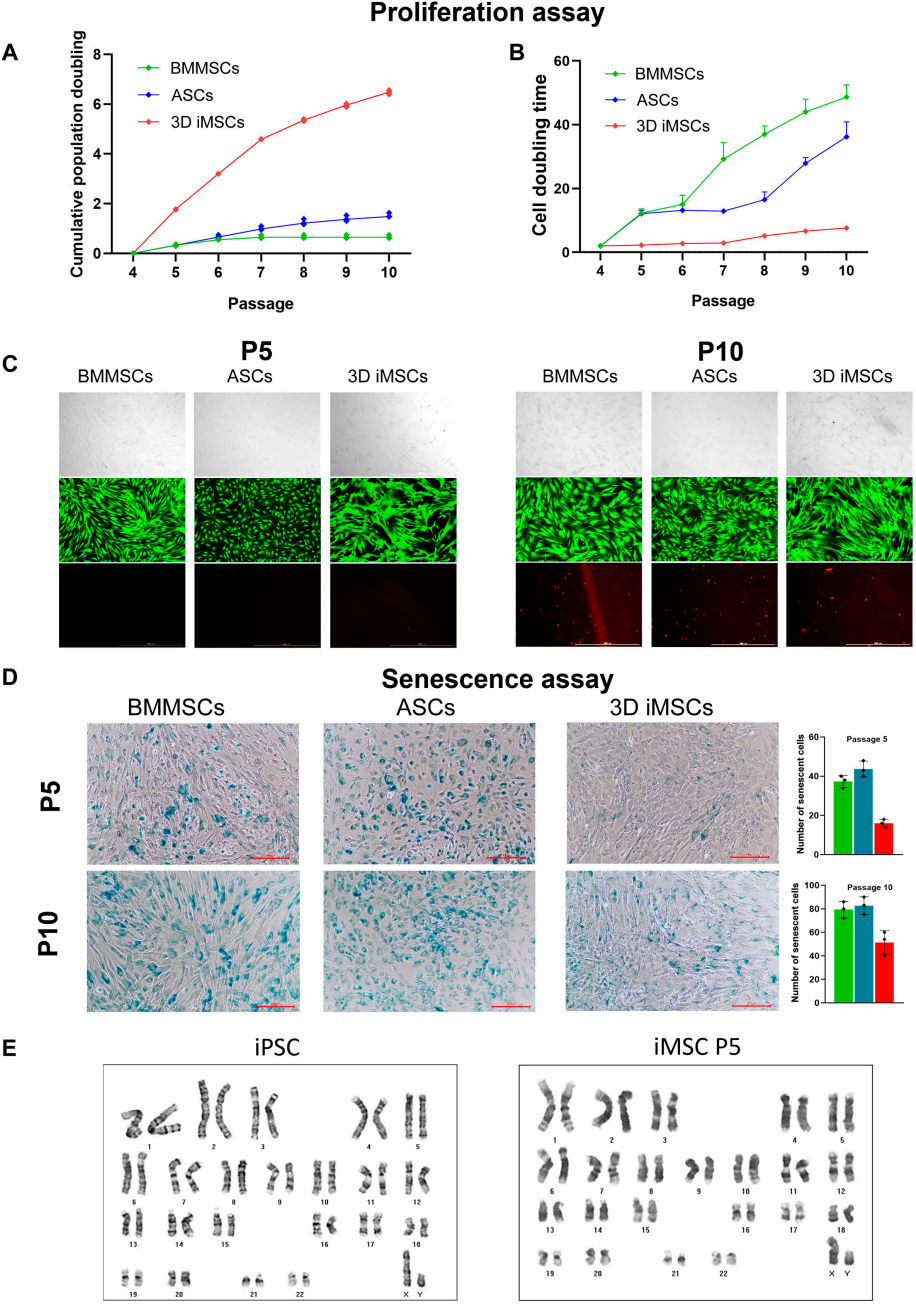
Proliferation and senescence of 3D iMSCs and adult-derived MSCs. (A) Cumulative population increases of BMMSCs, ASCs, and 3D iMSCs from passages 4 to 10. (B) Cell doubling times of BMMSCs, ASCs, and 3D iMSCs from passages 4 to 10. (C) Representative live/dead-stained BMMSCs, ASCs, and 3D iMSCs cultured for 2 d. Staining was performed at passages 5 and 10. (D) β-Galactosidase-stained MSCs at passages 5 and 10. The staining intensity was quantified by counting the number of blue cells. Scale bar = 200 μm. (E) Karyotype analysis of P25 iPSCs and P5 iMSCs. All data represent results from 3 independent experiments, each conducted in triplicate.

To further confirm the utility of our 3D differentiation approach, iMSCs were compared with other MSC types such as ASCs and BMMSCs. FACS analysis revealed that CD73, CD90, and CD44 were displayed on the surface of over 95% of iMSCs with further passage, similar to adult-derived MSCs (Fig. S6). Additionally, the absence of hematopoietic markers (CD34 and CD45) in these cells further indicated successful differentiation. The multipotency of MSCs was then assessed by qPCR, WB, and histological analyses. Alcian blue, alizarin red, and Oil Red O staining indicated chondrogenic, osteogenic, and adipogenic differentiation, respectively, in the obtained MSCs (Fig. 6A). These findings were further corroborated by WB and qPCR results (Fig. 6B and C). Additionally, iMSCs showed superior growth compared to ASCs and BMMSCs across the tested passages, as evidenced by their doubling times and cumulative proliferation (Fig. 7A and B). At passage 5, no dead cells were observed in any of the cell types. At passage 10, iMSCs showed fewer dead cells than other adult MSCs (Fig. 7C). Senescence assay based on β-galactosidase staining demonstrated that iMSCs are less senescent than ASCs and BMMSCs at both passages 5 and 10 (Fig. 7D). In addition to exhibiting higher proliferation rates, 3D iMSCs were found to be genetically stable with no detected chromosomal abnormality even after 5 passages, indicating its safety for clinical research (Fig 7E). Overall, these results demonstrated the robust multipotency of iMSCs and their improved cellular health compared to other MSCs, highlighting the vast potential of our approach for applications in regenerative medicine.

## Discussion

Over the years, differentiating embryonic stem cells or iPSCs into MSCs has been done in 2D systems that include GF treatment, co-culturing, genetic modification, or repetitive subculture [17,29,41–43]. However, 2D techniques have their own limitations, including the extended time required to generate MSCs, potential mutations from genetic modifications, and the risk of introducing unknown factors from animal cells in co-culture experiments, all of which may pose challenges in therapeutic applications [44,45].

In this study, we aimed to develop a novel, simple, and effective 3D culture method for generating MSCs from iPSCs. Previous research has shown that 3D cultures significantly enhance both differentiation and expansion compared to traditional 2D cultures [37,40,46,47]. To do this, we first established an optimal 2D culture conditions for iPSC to MSC differentiation that mimics the in vivo developmental process of MSC development via induction of mesoderm lineage. We screened BMP4, activin A, and Wnt3A to identify the most effective GF for inducing iPSC differentiation into mesodermal cells, with BMP4 emerging as the superior candidate.

Subsequent treatment of these cells with FGF2, which has been shown to induce MSCs from mesodermal cells and promote their proliferation, promoted the generation of MSC-like cells [12,31]. With the augmented proportion of MSC-like cells within the total cell population, a pure population of MSC-like cells was obtained after a few passages. Given that 3D culture conditions are more conducive to differentiation, specific GFs were strategically selected for stepwise differentiation. Mimicking human differentiation mechanisms is crucial in 3D differentiation; therefore, the size of EBs was assessed for its impact on mesoderm differentiation. Our findings indicated that 200-μm EBs are optimal for mesoderm induction.

EBs incorporating GelMPs have been shown to display reduced necrotic zones and improved GF distribution [35]. Accordingly, our findings also indicated that GelMPs mitigate necrotic zone formation by preventing dense cell clustering within EBs, thereby allowing improved oxygen and nutrient diffusion. Importantly, this also enabled improved delivery of GFs within EBs. We engineered GelMPs with varying concentrations of glutaraldehyde to facilitate the sequential release of BMP4 and FGF2 both critical for MSC differentiation. By altering the glutaraldehyde concentration, we were able to modulate the cross-linking density of the GelMPs, which directly influenced their swelling ratio, water content, and degradation rate. This, in turn, regulated their capacity to hold and release GFs.

Our experimental approach follows the natural stagewise differentiation of iPSCs into MSCs, mimicking in vivo development: initially differentiating into mesoderm through the BMP pathway, followed by MSC differentiation facilitated by FGF2 [8,18]. Consequently, the release of BMP4 for a shorter duration is required during the early stages, whereas FGF2 release is crucial at a later stage of differentiation. Designing GelMPs with distinct degradation rates and conjugating GFs accordingly ensures this sequential release and proper differentiation. Any alteration in this carefully orchestrated system can disrupt the sequential GF release, thereby negatively impacting iMSC generation. Thus, fast-degrading GelMPs were conjugated with BMP4 to initiate mesoderm induction, while slow-degrading GelMPs were functionalized with FGF2 for mesoderm differentiation into MSCs.

This approach notably increased MSC yield within a specified timeframe and simplified the process by eliminating the need for replenishing GFs daily, which are major advantages for scaling up production. Furthermore, the MSCs obtained exhibited characteristics similar to adult-derived MSCs, such as BMMSCs and ASCs. Additionally, the derivation of MSCs from iPSCs imparts traits such as high proliferation rates and reduced senescence, facilitating the large-scale production of iMSCs. While these iMSCs demonstrate comparable chondrogenic and osteogenic potential, they exhibit lower adipogenic differentiation. This variation may be attributable to the specific iPSC cell line used, as the source of the iPSC can significantly influence the differentiation capabilities of iMSCs [48]. These cells, which share characteristics with MSCs, do not contain any chromosomal abnormalities (GTG karyotyping), indicating that the cells are safe for clinical use. Overall, the simplicity and effectiveness of our proposed culture technique highlight its potential for large-scale production and therapeutic applications, paving the way for advancements in regenerative medicine.

While our differentiation system is innovative and simple, there is a considerable potential for further advancements. This system has laid the groundwork for MSC differentiation using GelMPs to deliver GFs. However, the reliance on a single iPSC cell line in our research may influence the differentiation outcomes. To address this, we recommend testing the system with various iPSC cell lines. Additionally, emerging technologies offer exciting opportunities for enhancement. For instance, oxygen nanobubbles represent an emerging nanoparticle technology primarily used to deliver oxygen to target areas of hypoxia [49,50]. Since our paper claims that the use of 3D spheroids induces necrotic zones possibly due to hypoxic conditions at the core of the spheroids, it would be beneficial to utilize nanobubbles loaded with MSC-specific GFs. This could enhance the efficiency of iPSC-to-MSC differentiation by providing both oxygen and essential GFs in the core of the EBs. Other potential modifications could include the integration of 3D printing, the use of smart biomaterials, and the incorporation of diverse differentiation agents, such as additional GFs, small molecules, messenger RNA, and small interfering RNA. Future research should aim to fully optimize the system within a 3D environment, allowing for precise tuning of variables like GF concentration and incubation time. These improvements could potentially enhance the system’s efficiency, scalability, and applicability across various biomedical fields.

## Conclusion

Overall, this study introduced an innovative one-step iMSC production method incorporating degradable GelMPs for controlled GF supplementation. This approach enabled sequential and improved delivery of BMP4 and FGF2 within spheroids, promoting homogeneous and enhanced cell differentiation compared to soluble GF treatment while preventing necrotic zone formation. This straightforward and effective method holds great potential for large-scale MSC production, offering tremendous opportunities in the field of regenerative medicine.

## Data Availability

The authors declare that all data supporting the findings of this study are available within the paper and its Supplementary Materials files.
