# Supplementary material for: Derivation of Mesenchymal Stem Cells through Sequential Presentation of Growth Factors via Gelatin Microparticles in Pluripotent Stem Cell Spheroids
Source: Biomater Res. 2025 Apr 29;29:0184. doi: 10.34133/bmr.0184 (PMC12038162; doi:10.34133/bmr.0184)
Supplement: Supplementary 1 — Figs. S1 to S6 [file bmr.0184.f1.docx]

**Derivation of mesenchymal stem cells through sequential presentation of growth factors via gelatin microparticles in pluripotent stem cell spheroids**

**Supplementary Figures:**


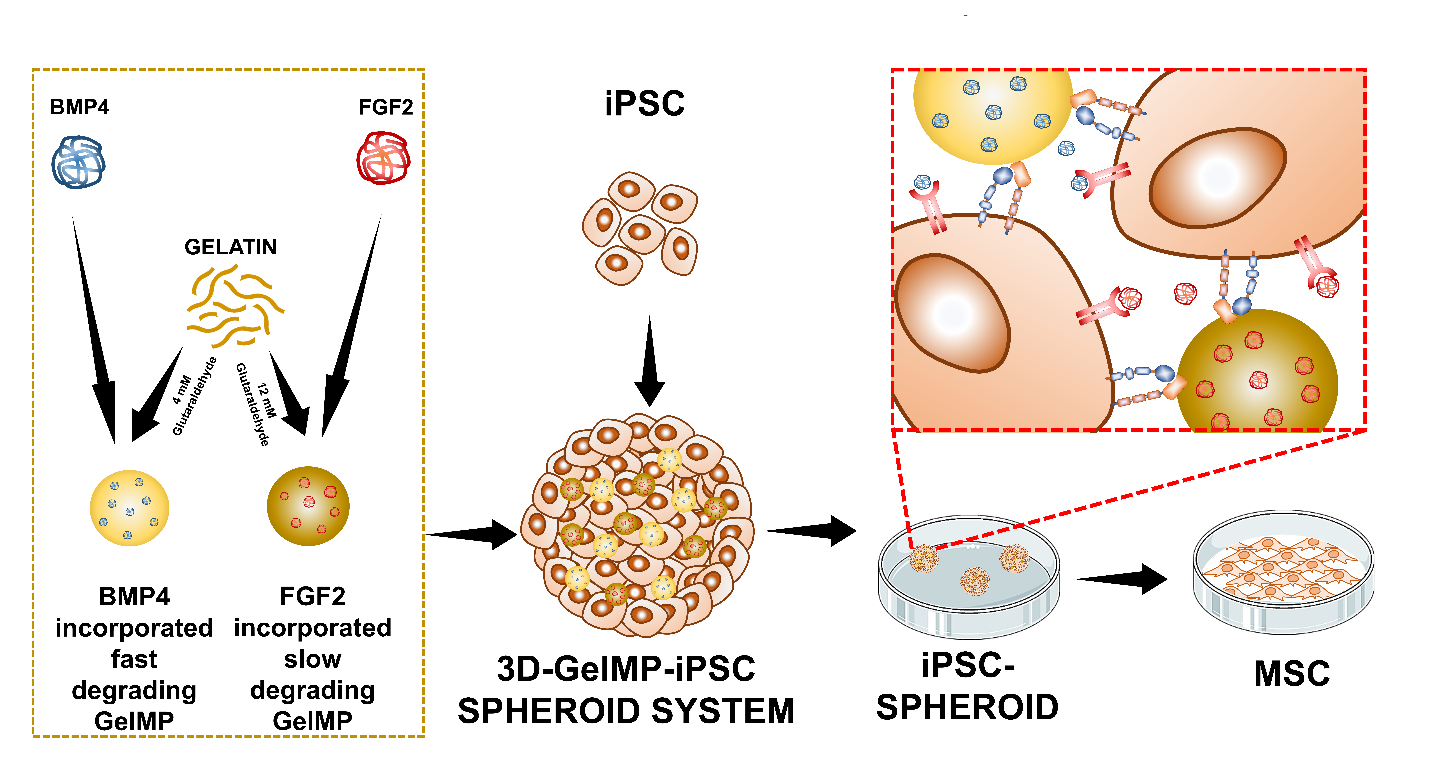


**Supplementary Figure 1. A three-dimensional (3D)** **spheroid culture system employing dual-release gelatin microparticles to facilitate enhanced iPSC-to-MSC differentiation.** This study introduces a 3D spheroid culture system based on dual growth factor-delivering gelatin microparticles (GelMPs) that enhances the differentiation of induced pluripotent stem cells (iPSCs) into mesenchymal stem cells (MSCs). The fast-releasing GelMPs are conjugated with bone morphogenic protein 4 (BMP4), while the slow-releasing GelMPs are conjugated with fibroblast growth factor 2 (FGF2). This enables controlled and sequential release of the respective growth factors for optimal differentiation. The application of these functionalized GelMPs in embryoid bodies (iPSC spheroids) significantly improves MSC differentiation, demonstrating its vast potential in advancing regenerative medicine.


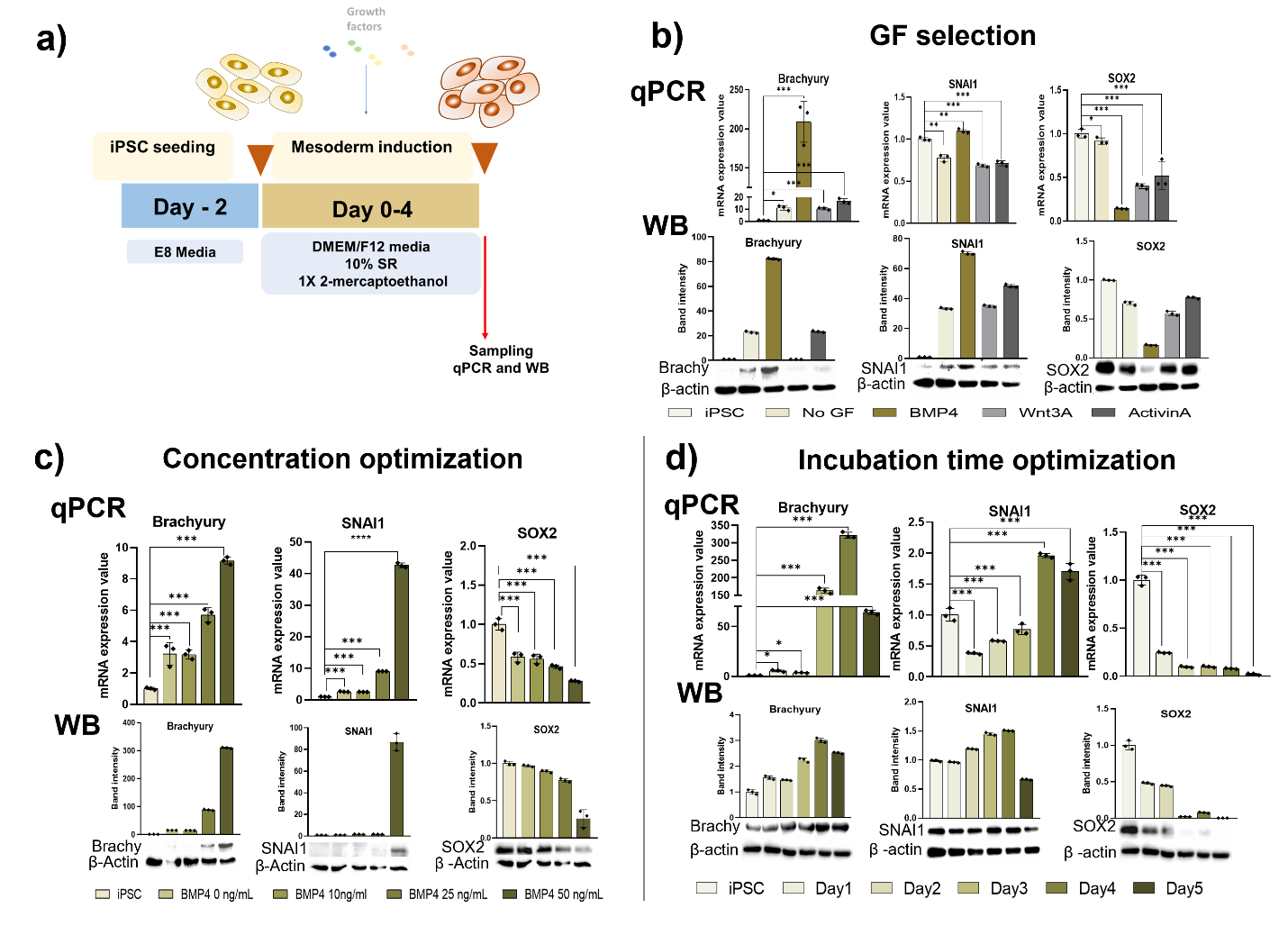


**Supplementary Figure 2. Growth factor selection and optimization for iPSC to mesoderm induction.** GF selection and optimization for iPSC differentiation into mesoderm. **a)** Schematic diagram of mesoderm induction. qPCR (top panel) and WB (bottom panel) analyses of mesoderm markers (Brachyury and SNAI1) and pluripotency markers SOX2 for **b)** GF selection**, c)** for concentration optimization, and **d)** incubation time optimization. The qPCR data was normalized to 18S. The WB data was normalized to β-actin. All data represent results from three independent experiments, each conducted in triplicate. The data are presented as mean ± s.d. (ns = not significant, * *p* < 0.05, ** *p* < 0.01, *** *p* < 0.001; ****, *p* < 0.0001). Individual data points and significance levels are indicated in graphs.


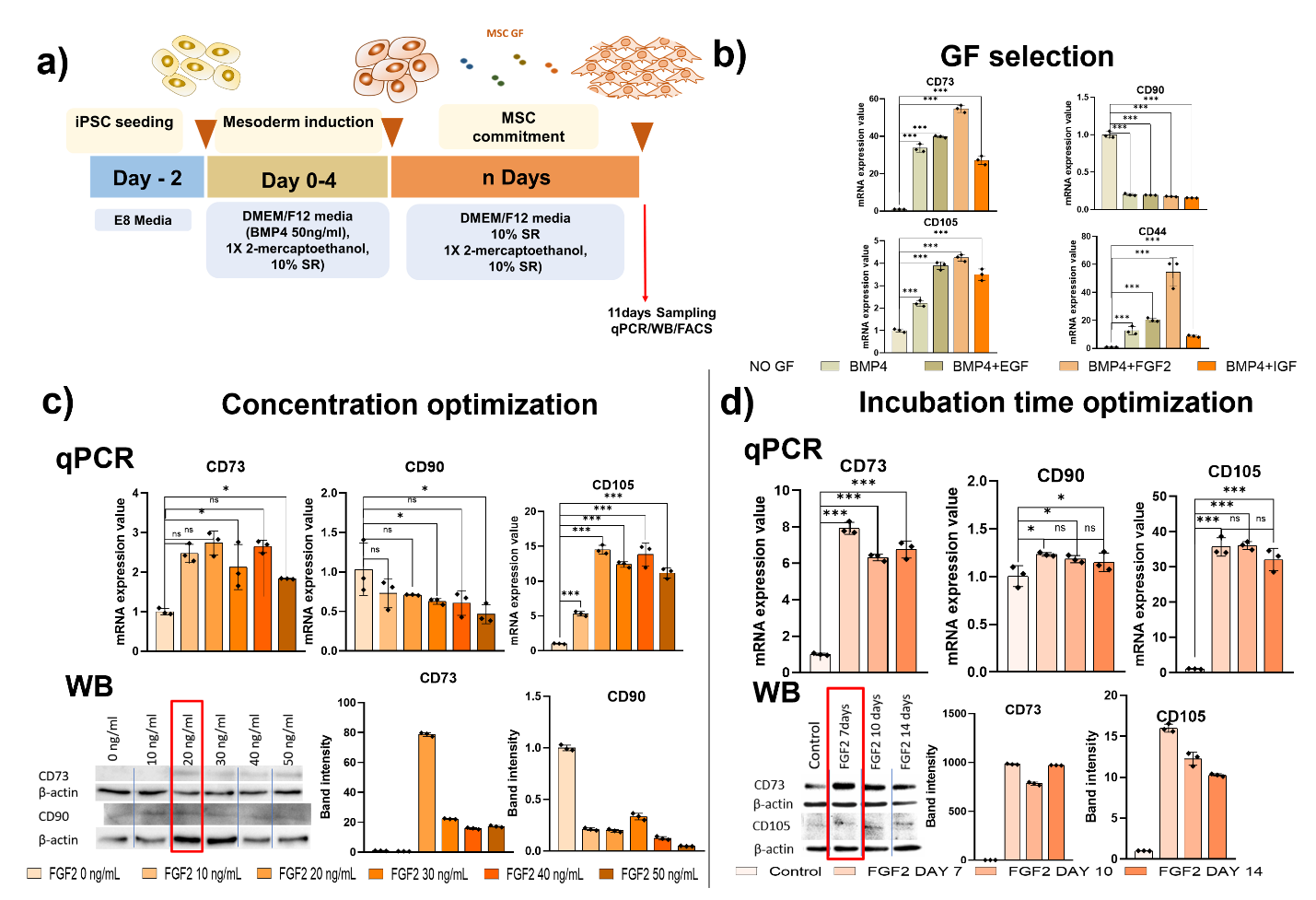


**Supplementary Figure 3**. **Growth factor selection and optimization for mesoderm to MSC differentiation.** GF selection and optimization for mesoderm to MSC differentiation. **a)** Schematic illustration depicting mesoderm to MSC differentiation. **b)** qPCR analysis of MSC markers (CD73, CD90, CD105, and CD44) at day 11 for GF selection. **c)** qPCR (top panel) and WB (bottom panel) analyses of MSC markers (CD73, CD90, CD105, and/or CD44) at day 11 for FGF concentration optimization. **d)** qPCR (top panel) and WB (bottom panel) analyses of MSC markers (CD73, CD90, and/or CD105) for FGF2 incubation time optimization. The qPCR data was normalized to 18S. The WB data was normalized to β-actin. All data represent results from three independent experiments, each conducted in triplicate. The data are presented as mean ± s.d. (ns = not significant, * *p* < 0.05, ** *p* < 0.01, *** *p* < 0.001; ****, *p* < 0.0001). Individual data points and significance levels are indicated in graphs.


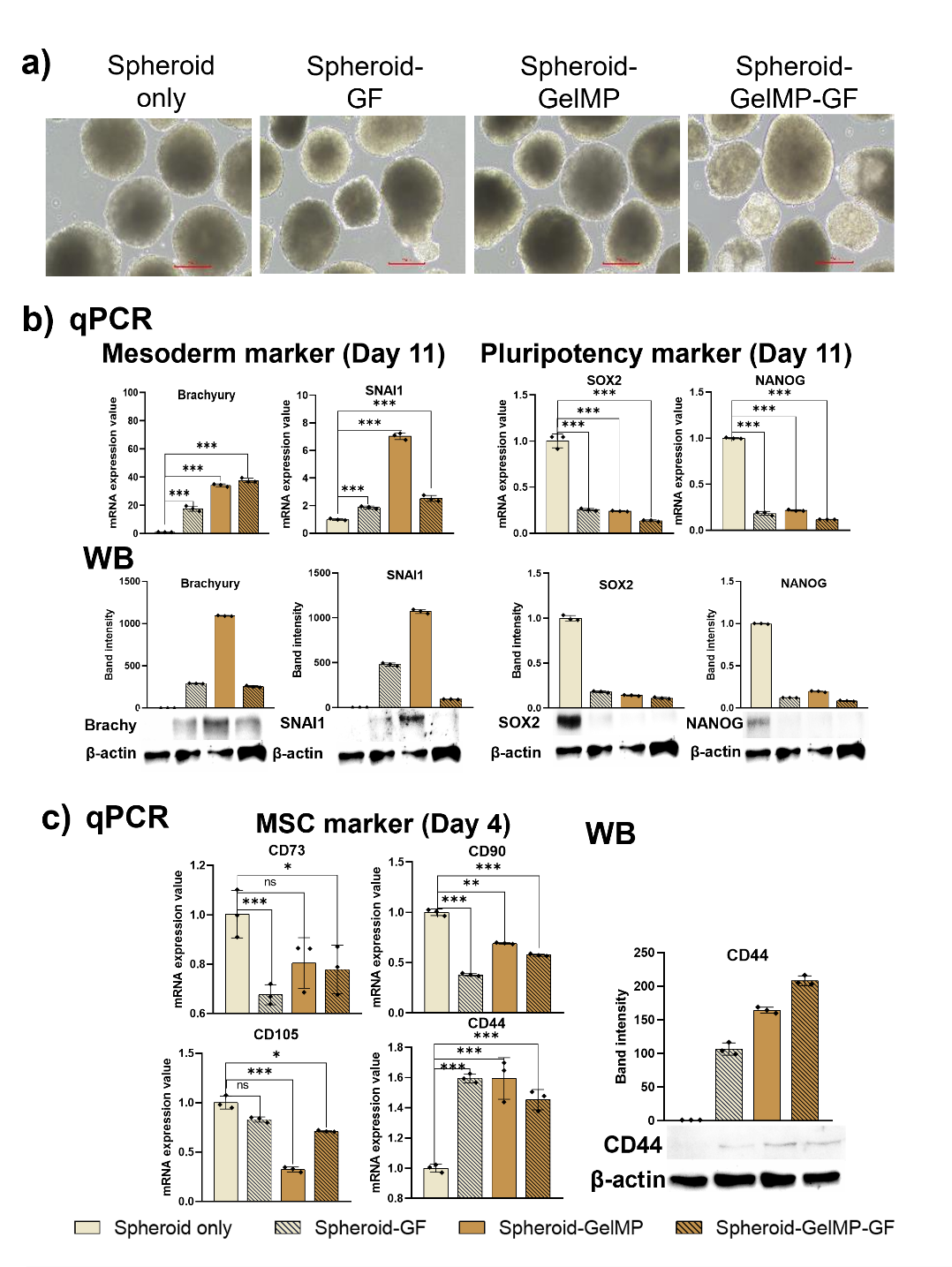


**Supplementary Figure 4. Effect of soluble and GF-GelMP mediated 3D differentiation of iPSC in MSC marker generation.** The effects of soluble and conjugated GFs on 3D differentiation of iPSCs into MSCs. **a)** Images of spheroid-only, spheroid-GF, spheroid-GelMP, and spheroid-GelMP-GF cultured for 11 days. **b)** qPCR (top panel) and WB (bottom panel) analyses of mesoderm markers (Brachyury and SNAI1) and pluripotent markers (SOX2 and NANOG) at day 11. **c)** qPCR (left panel) and WB (right panel) analyses of MSC markers (CD73, CD105, and/or CD44) at day 4. The qPCR data was normalized to 18S. The WB data was normalized to β-actin. All data represent results from three independent experiments, each conducted in triplicate. The data are presented as mean ± s.d. (ns = not significant, * *p* < 0.05, ** *p* < 0.01, *** *p* < 0.001; ****, *p* < 0.0001). Individual data points and significance levels are indicated in graphs.


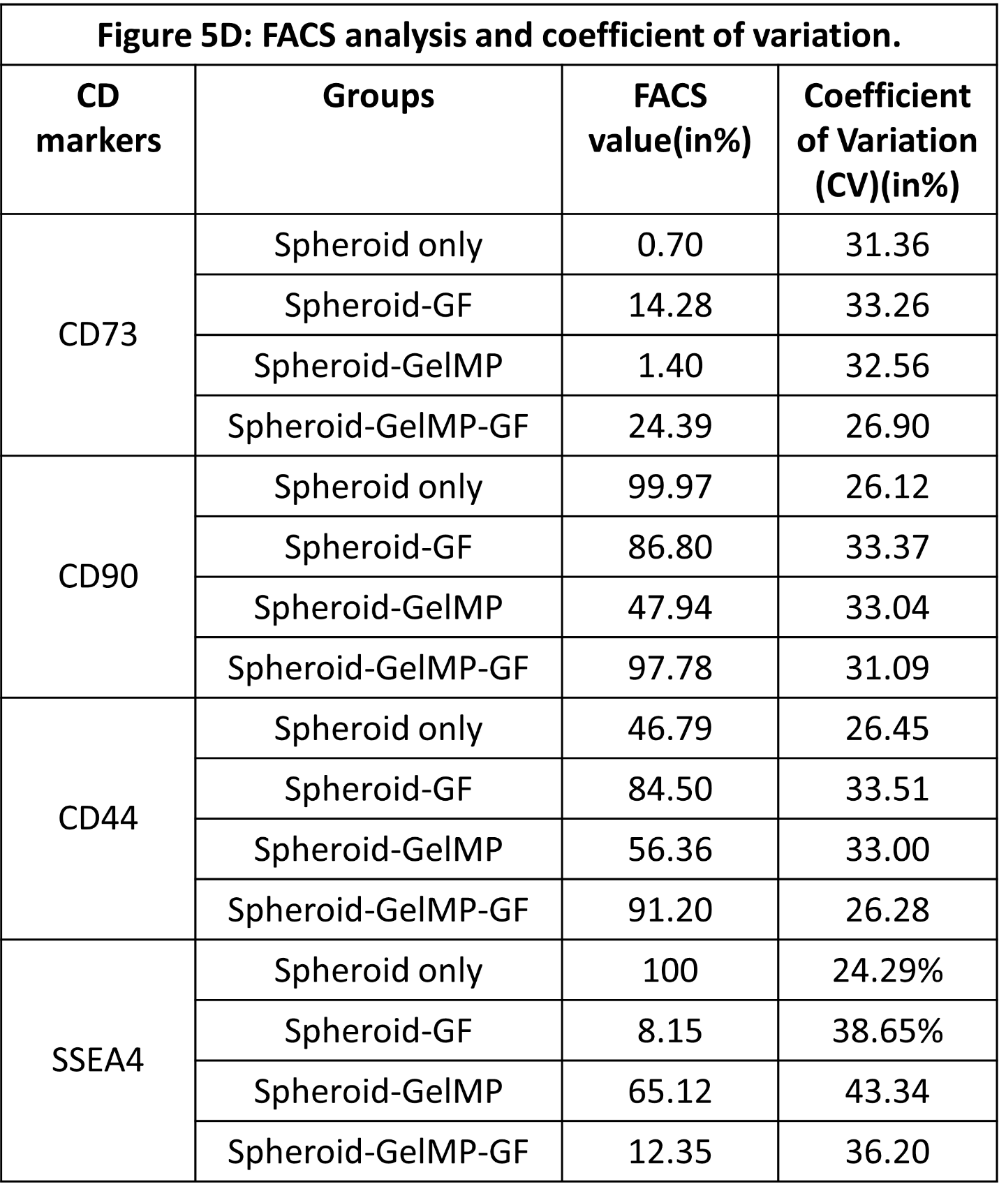


**Supplementary Figure 5. Values of FACS analysis and Coefficient of variation for FACS results of Figure 5D.**


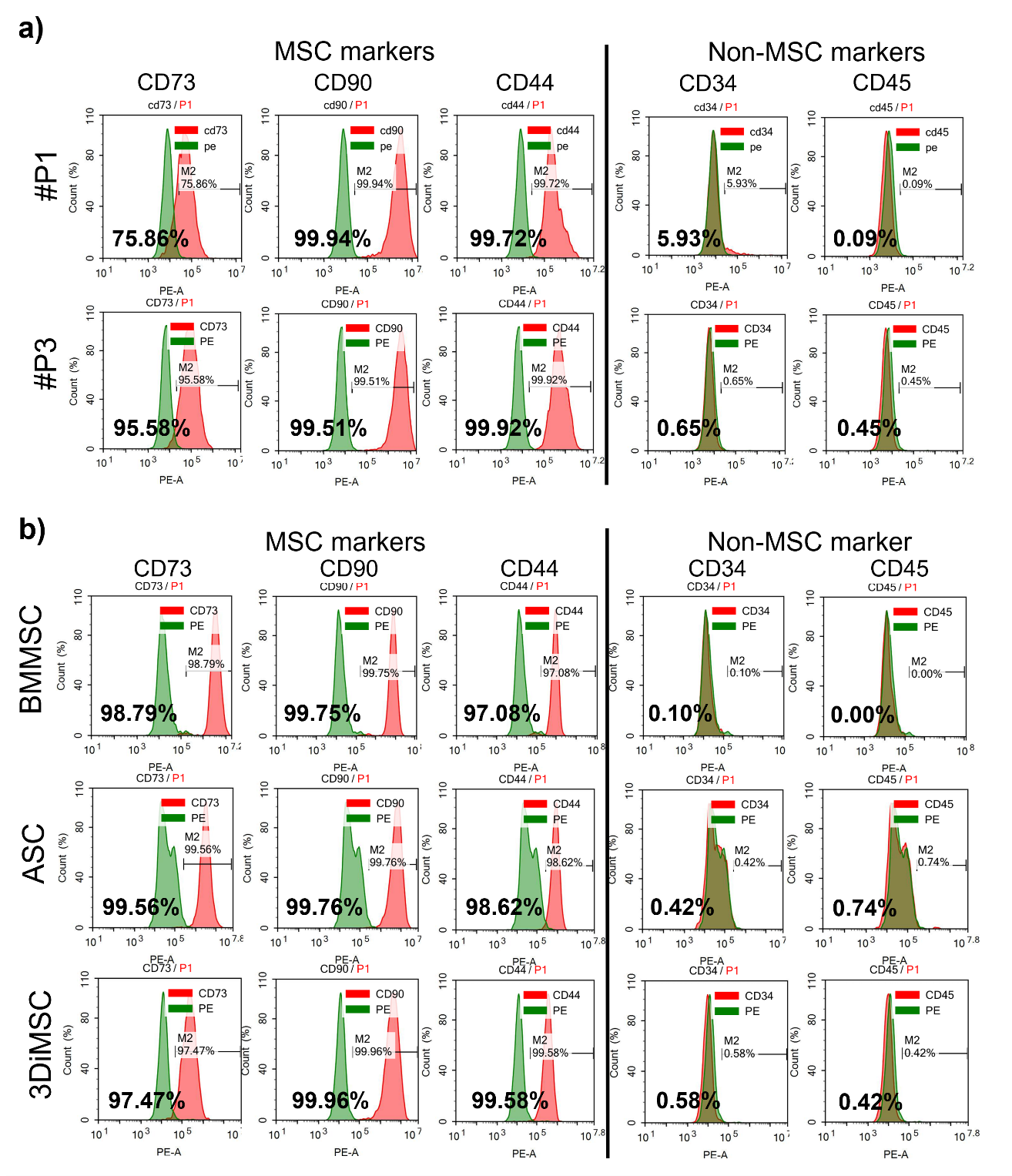


**Supplementary Figure 6. FACS analysis of 3D-iMSC.** FACS analysis of 3D iMSCs. **a)** FACS plots of cells at passages 1 and 3 for MSC markers (CD73, CD90, and CD44) and non-MSC markers (CD34 and CD45). **b)** FACS plots of BMMSCs, ASCs, and 3D iMSCs at passage 5 for MSC markers (CD73, CD90, and CD44) and non-MSC markers (CD34 and CD45). All data represent results from three independent experiments, each conducted in triplicate.
